# Clinical and Environmental Surveillance for *Vibrio cholerae* in Resource Constrained Areas: Application during a 1-Year Surveillance in the Far North Region of Cameroon

**DOI:** 10.4269/ajtmh.15-0496

**Published:** 2016-03-02

**Authors:** Amanda K. Debes, Jerome Ateudjieu, Etienne Guenou, Walter Ebile, Isaac Tadzong Sonkoua, Anthony Chebe Njimbia, Peter Steinwald, Malathi Ram, David A. Sack

**Affiliations:** Johns Hopkins University Bloomberg School of Public Health, Baltimore, Maryland; Department of Biomedical Sciences, University of Dschang, Dschang, Cameroon; Meilleur Accès aux Soins de Santé (M.A. SANTE), Yaoundé, Cameroon; Clinical Research Unit, Division of Health Operations Research, Ministry of Public Health, Yaoundé, Cameroon

## Abstract

Biological confirmation of the presence of *Vibrio cholerae* in clinical and environmental samples is often constrained due to resource- and labor-intensive gold standard methods. To develop low-cost, simple, and sustainable surveillance techniques, we modified previously published specimen sampling and culture techniques and applied the use of enriched dipstick testing in conjunction with the use of filter paper for DNA specimen preservation during clinical and environmental surveillance in the Far North of Cameroon from August 2013 to October 2014. The enriched dipstick methodology during routine use in a remote setting demonstrated a specificity of 99.8% compared with polymerase chain reaction (PCR). The novel application of filter paper as a preservation method for cholera DNA specimens reduced the need for cold chain storage and allowed for PCR characterization and confirmation of *V. cholerae*. The application of basic technologies such as the enriched dipstick, the use of simplified gauze filtration for environmental sample collection, and the use of filter paper for sample preservation enabled early case identification with reduced logistics and supply cost while reporting minimal false-positive results. Simplified laboratory and epidemiological methodologies can improve the feasibility of cholera surveillance in rural and resource-constrained areas, facilitating early case detection and rapid response implementation.

## Introduction

Accurate and reliable infectious disease burden data are often minimally available or nonexistent in developing countries. Cholera disease burden data are similarly deficient due to lack of surveillance in endemic areas as well as a fear of economic repercussions from reporting outbreaks, ultimately resulting in reduced reporting at district and national levels.^1^ Reporting is also constrained by logistics and costs associated with transport of samples to a reliable microbiology laboratory. Of the 47 countries that reported cholera cases and deaths to the World Health Organization (WHO) in 2013, 22 were in Africa.^2^ In spite of reporting the largest cholera disease burden in the world, there continues to be a poor understanding of the disease patterns in much of Africa because of constraints in detecting and confirming cholera cases. In fact, most cases that are reported are based on clinical case definitions without laboratory confirmation; significantly more cases remain undetected and unreported every year because of the inability to confirm a case. This presents a challenge for proper disease surveillance and laboratory capacity requirements, especially in remote, rural areas and contributes to a lack of understanding of cholera disease burden.

Although population-based surveillance provides the most accurate information for disease burden, it is too resource intensive for most cholera settings. Another option, sentinel surveillance, may be used in resource-limited settings to monitor a disease of interest at a specific site. This method requires fewer resources than population-based surveillance to identify the disease if the disease of interest is present.^3^ However, since cholera tends to be sporadic, sentinel surveillance may not provide representative data of the complete disease burden. Despite its comparative economic advantages over population-based surveillance, sentinel surveillance can also be difficult to sustain in low-resource settings given other health and political priorities and the lack of funding to support a concerted effort to track a single disease.

A modified approach to sentinel surveillance has been successfully implemented in Bangladesh where cholera has a more consistent seasonal pattern. Since 1997, environmental and clinical surveillance has been conducted in different locations in Bangladesh on a reduced schedule of surveillance for three successive days every 15 days. This strategy has shown that a simplified surveillance methodology can aide in documenting disease burden with reduced operational costs, while providing data to better understand the seasonality and transmission patterns of the disease.^4^

Diagnostic confirmation of *Vibrio cholerae* infection is challenging in rural and resource-limited settings. The accepted method of *V. cholerae* diagnosis is through culture confirmation, requiring well-equipped laboratories and trained laboratory personnel. This method is time consuming, resource intensive, and prohibitively expensive for these settings. As a result, cholera is often only diagnosed using a clinical definition recommended by the WHO, which classifies cholera case as a patient aged 5 years or more who develops severe dehydration or dies from acute watery diarrhea.^5^,^6^ More often, cholera is not diagnosed, and the patient is treated without identifying the etiologic agent. Commercially available dipstick tests, such as Crystal VC^™^ (Arkray Healthcare Pvt Ltd., Surat, India), allow for rapid diagnosis. Unfortunately, previous studies have reported low specificity when using the kits with direct testing of stool samples per recommendation by the package insert.^7^–^9^ We have applied a modified approach to using the dipstick in both stool^10^–^12^ and environmental specimens^13^ that has previously demonstrated improved specificity of the dipstick.

In this study, we demonstrate the successful implementation of sentinel surveillance using low-cost and rapid laboratory diagnostics in a low-resource setting. The simplified methodology facilitates concurrent environmental and clinical surveillance to provide crucial disease burden information, determine hot spots for cholera activity, improve understanding of cholera transmission patterns, and provide tools for early disease detection. Further, we report the novel use of filter paper technology for preservation of specimens for DNA extraction and molecular processing. Although culture methods are often considered to be the gold standard, they are not 100% sensitive, particularly if the patient has self-medicated with antibiotics before seeking treatment at a health facility. Therefore, for our study, we considered a positive polymerase chain reaction (PCR) to be the model gold standard of detection.

## Materials and Methods

### Study design.

Cameroon has a population of approximately 20 million people, divided into 10 regions with varied geographies and diverse climate zones (25). The Far North region of Cameroon (FNC) is located in the Sahel desert and is composed of a predominantly rural population, of which less than half have access to improved drinking water.^14^ The incidence of cholera disease is becoming increasingly common in Cameroon, particularly in the FNC where 10 outbreaks occurred from 1996 to 2014. Of all of these, the outbreak in 2010 was the most severe with almost 10,000 cases and 599 deaths (a 6.37% case fatality rate).^15^ These numbers likely underestimate the true burden of disease due to lack of surveillance and laboratory capacity.

Diarrhea surveillance was established at seven local health facilities in the FNC, in and around Lake Chad, including Kousseri Regional Hospital (Kousseri Health District), Mada District Hospital (Mada Health District), Ngouma Integrated Health Center (Makary Health District), Maltam Integrated Health Center (Goulfey Health District), Blangoua Subdivisional Medical Center (SMC) (Mada Health District), Darak SMC (Mada Health District), and Naga Integrated Health Center (Mada Health District). These sites were selected as being geographically representative of the Cameroonian health facilities near Lake Chad. The selected sentinel sites implemented a simplified sampling methodology in which each of the seven sentinel site district health facilities enrolled consenting diarrheal cases of any age into the study for a 3-day period every 15 days. During the intervening days, any suspected cholera patient meeting the WHO definition of cholera was also requested to be enrolled in the study. Concurrently, environmental sampling was conducted 1 day of every 15 days. Study enrollment began in August 2013. Subjects with bloody diarrhea and diarrhea lasting more than 7 days were excluded.

### Clinical surveillance.

**Figure 1.**
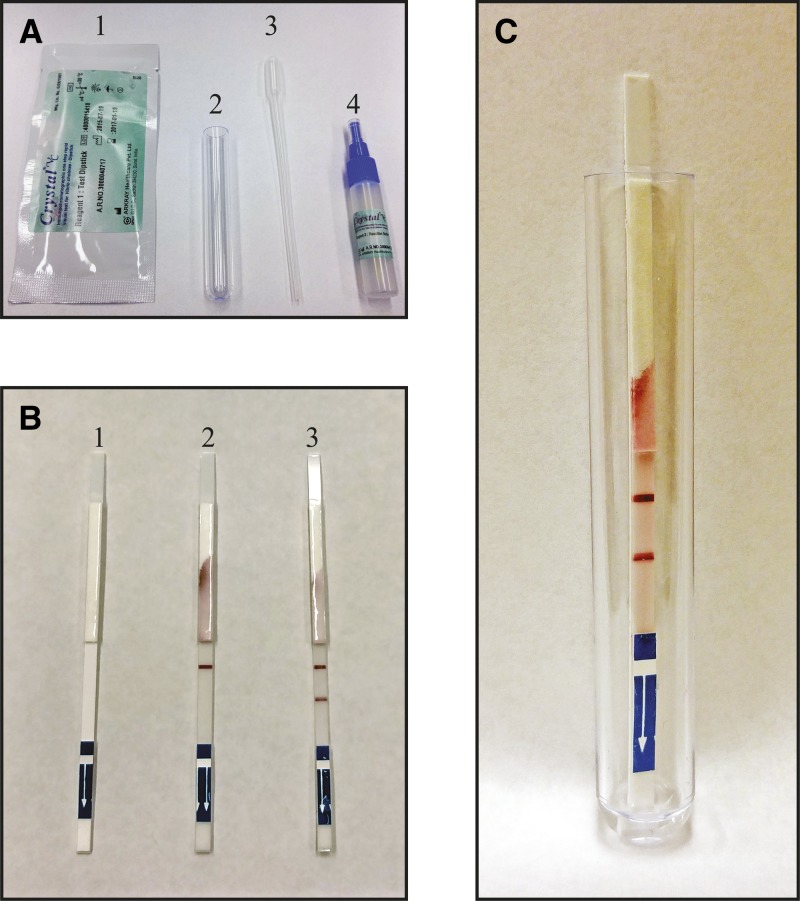
Crystal VC kit. (**A**) 1) The dipstick is provided in an individualized, humidity controlled package; 2) a clean test tube is provided in the kit for each dipstick tested; 3) a Pasteur pipette is provided in the kit for each specimen to be transferred; and 4) a specimen processing vial containing enrichment media. (**B**) 1) A clean dipstick; 2) a negative dipstick showing clearly the control line only; 3) a *Vibrio cholerae* O1–positive dipstick showing both the control line and the O1-positive line. (**C**) A *V. cholerae* O1–positive dipstick as it appears when testing in the kit-provided test tube; note that only ∼200 μL of specimen should be added to the tube as indicated by the arrow.

Fecal specimens were collected from consenting diarrhea subjects and screened for *V. cholerae* O1 and O139 using an enhanced dipstick method (Figure 1A

; Crystal VC) in which the specimen is tested via dipstick after incubation for 6–8 hours in alkaline peptone water (APW; in grams per liter: peptone, 10 g; sodium chloride, 10 g, pH 9.0–9.2). A positive result appears as two pink lines on the dipstick; the upper line being the control band and the lower band being the lipopolysaccharide specific to serogroup O1 positive band (Figure 1B and C). APW-enriched samples that tested positive, as well as a minimum of 10% of negative clinical samples, were inoculated into Cary-Blair transport media (Becton Dickinson and Co., Franklin Lakes, NJ) for storage and transport to the central reference laboratory in the Kousseri health facility for microbiological confirmation. Using a Pasteur pipette, one to two drops of the enriched APW specimen were spotted onto a Whatman 903 211 Protein Saver Card (GE Healthcare Ltd., Forest Farm, Cardiff, UK) and allowed to air-dry. Filter paper was evaluated for viable vibrios after overnight drying at room temperature, and all filter paper specimens tested were culture negative. Filter paper specimens were stored in individual plastic bags at room temperature until DNA extraction and PCR processing were implemented at a later date. The dipsticks and the preparation of the Protein Saver Cards were carried out in the individual hospitals by nurses, and the filter paper samples were then sent to the central laboratory in Kousseri for preservation. Dipsticks, conical tubes containing APW-enriched specimens, and test tubes were treated as biohazardous material and disinfected before disposal. A detailed training manual for the detection of *V. cholerae* O1 was created by several of the collaborating authors during the study period, available at stopcholera.org.^16^

### Environmental surveillance.

**Figure 2.**
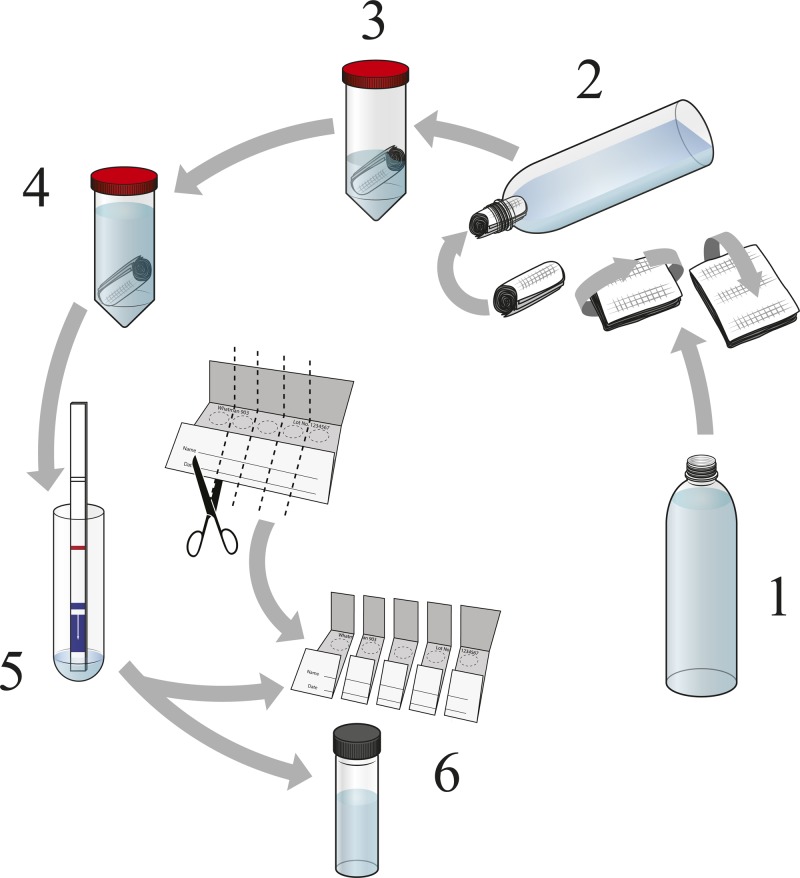
Procedure for detecting *Vibrio cholerae* O1 from environmental source using enriched dipstick method. Figure 2 is a pictorial of the steps used to collect and assess *V. cholerae* O1 from environmental samples. Step 1 is to collect 2–3 L water from environmental sampling site. Step 2 shows that one should take medical gauze and fold it, then roll it into a tube to be placed into the mouth of the funnel device, ensuring that the gauze fits snug into the mouth of the funnel so that it will not be displaced when funneling water. The funnel can be a plastic bottle with the base cutoff or, if available, a funnel. Once the gauze filter is in place in the mouth of the funnel, then 2–3 L water is filtered through the funnel. Step 3 is once the water has all been filtered through the funnel, remove the gauze filter and place into a 50-mL conical tube containing 20 mL of 2× alkaline peptone water (APW). The APW is 2× to offset any water retained in the filter, which would reduce the concentration of the APW. Step 4 is to incubate the gauze in the 2× APW solution for ∼24 hours at ∼37°C (or between 25°C and 40°C room temperature [RT] if incubator is not available). Step 5 is to use the Pasteur pipette included in the Crystal VC dipstick kit and transfer ∼200 μL of the enriched media to the test tube provided with the kit. Then insert a new dipstick into the solution and allow to incubate at RT for 15 minutes. Read the dipstick at 15 minutes to determine if it is positive or negative (see Figure 1B above). Step 6 is to preserve the specimen in Cary Blair and on precut individual Whatman filter paper cards; all positives and a 10% sample of negatives were preserved on Cary Blair and filter paper for culture confirmation.

Surface water samples were collected once every 15 days from six sites near each of the sentinel health facilities (total of 42 sites) to be tested for the presence of *V. cholerae* O1 and O139. In a plastic jar, 3 L of the surface water was collected and then filtered through sterile gauze. The gauze (∼20 mL) was then combined with equivalent volume (in mL) 2× APW (final concentration of 1× APW) and incubated for 24 hours (±2 hours). The overnight enriched solution was tested using the dipstick. All positive and selected negative environmental samples were inoculated into Cary-Blair transport media for storage until transported to the central reference laboratory for microbiological confirmation (Figure 2

). Using a Pasteur pipette, one to two drops of the APW-enriched specimens were preserved on a Whatman 903 Protein Saver Cards and allowed to air-dry. As with the clinical specimens, filter paper specimens were stored in individual plastic bags at room temperature until DNA extraction. The processing of the environmental samples was carried out by technicians at the individual hospitals. Enriched specimens were decontaminated following the protocol for biohazardous material; dipsticks, conical tubes containing APW-enriched specimens, and test tubes were treated as biohazardous material and disinfected before disposal.

### Microbiological testing at the central laboratory.

All positive clinical and environmental specimens and selected negative specimens were sent at routine (biweekly) intervals to the central laboratory in Kousseri for confirmation. The specimens were streaked directly onto thiosulfate citrate bile salt sucrose (TCBS; Becton Dickinson and Co., Franklin Lakes, NJ) agar and incubated for 24 hours at 37°C. Immediately after inoculating, the first TCBS plate, a pre-labeled APW vial was inoculated with the specimen and incubated for 6 hours at room temperature. After 6-hour incubation, a second TCBS plate was inoculated with the enriched specimen and incubated for 24 hours at 37°C. After 24-hour incubation, any cholera-like colonies were selected with a sterile loop, resuspended in one to two drops of phosphate-buffered saline (PBS), and tested via dipstick. All dipstick-positive cultures, as well as any cultures considered cholera suspect (demonstrating the morphology of a cholera colony), were preserved in T1N1 agar (1% tryptone and 1% NaCl) for further testing.

### DNA extraction.

The dried filter paper samples were sent to Baltimore, MD, for PCR analysis. DNA extractions were performed using methods similar to those previously published.^17^ Each dried filter paper specimen was excised using sterile scissors and placed into a pre-labeled tube. Of sterile 1× PBS, 1 mL was added to each sample tube and incubated for 10 minutes at room temperature. One mL of sterile 1× PBS was then added to each sample, and immediately centrifuged (14,000 × g for 2 min) and the supernatant discarded. To each sample, 1 mL sterile 1× PBS was added, and then the sample was immediately centrifuged (14,000 × *g* for 2 minutes) and the supernatant was discarded. Subsequently, 150 μL of a 2% (w/v) Chelex-100 solution (Bio-Rad, Hercules, CA; catalog no. 1422832) followed by 50 μL of sterile water was added to each sample. The samples were placed in a heating block at 100°C for 8 minutes and then centrifuged (14,000 × *g* for 2 minutes). The supernatant was transferred to a new microcentrifuge tube and either stored at −20°C or used in a PCR amplification reaction. The quantity and quality of extracted DNA was determined using an ultraviolet (UV) spectrophotometer (NanoDrop 2000, Thermo Scientific, Waltham, MA).

### Polymerase chain reaction.

As an initial screening step, a multiplex PCR amplification was performed to determine the presence of three *Vibrio* species in the DNA sample by targeting the *toxR* genes of *Vibrio vulnificus*, *Vibrio parahaemolyticus*, and *V. cholerae*. Following the previously described PCR conditions, the universal forward primer UtoxF was used in combination with species-specific primers (Table 1) VvtoxR, VptoxR, and VctoxR to amplify products of 297, 640, and 435 bp, respectively.^18^ If *V. cholerae* was identified in the sample, a second multiplex PCR was then performed to differentiate nontoxigenic and toxigenic *V. cholerae*. The multiplex was performed as described by Nandi and others with 15 pmol of primers targeting an outer membrane protein gene, *OmpW*, which is a unique gene conserved in the *V. cholerae* genome, in combination with primers targeting cholera toxin A (*ctxA*) gene (Table 1) at a concentration of 6.2 pmol.^19^ The amplified PCR products for *OmpW* and *ctxA* were 588 and 301 bp, respectively. Subsequently, all *V. cholerae*-positive specimens were tested to determine if they belonged to serogroup O1 or O139, regardless of their toxigenic nature. This multiplex targets unique regions in the *rfb* gene specific for the O1 and O139 serogroups. PCRs were conducted following methods described by Hoshino and others^20^ with PCR optimization including an increase in primer concentration to 2 μM for both O1- and O139-specific primers (Table 1). On any negative samples, 16S rDNA PCR was performed to confirm DNA preservation and extraction techniques by applying methods described by Hasan and others.^21^ The primers, 6968-GC (V6/V8) and L1401 (Table 1) selectively amplify the variable regions V6, V7, and V8 of 16S rDNA genes from most bacteria producing a 457-bp amplicon. The presence of PCR products was determined using standard 2% agarose gel electrophoresis containing the intercalating agent ethidium bromide and visualized under UV light.

### Statistical methods.

Clinical and environmental samples collected between August 2013 and October 2014 were used for these analyses. Descriptive statistics were used to plot the number of confirmed cholera and diarrhea cases comparing intensive versus routine surveillance for each study area over the first year of the study. The environmental surveillance data included the type of water source (pond, river, ditch, well, sewage drain, or lake) and the date of collection. The occurrence of vibrios in the environment was compared over time by date, facility, and water source. We estimated the significance of *V. cholerae* non-O1 detection over the first year of the study, taking into account the differing water sources, follow-up visits, and within-facility clustering of detection. We used generalized estimating equations with a log link function and an ar1 correlation matrix to account for clustering at the facility level. Statistical analyses were conducted with Stata 13 (StataCorp LP, College Station, TX).^22^

## Results

**Figure 3.**
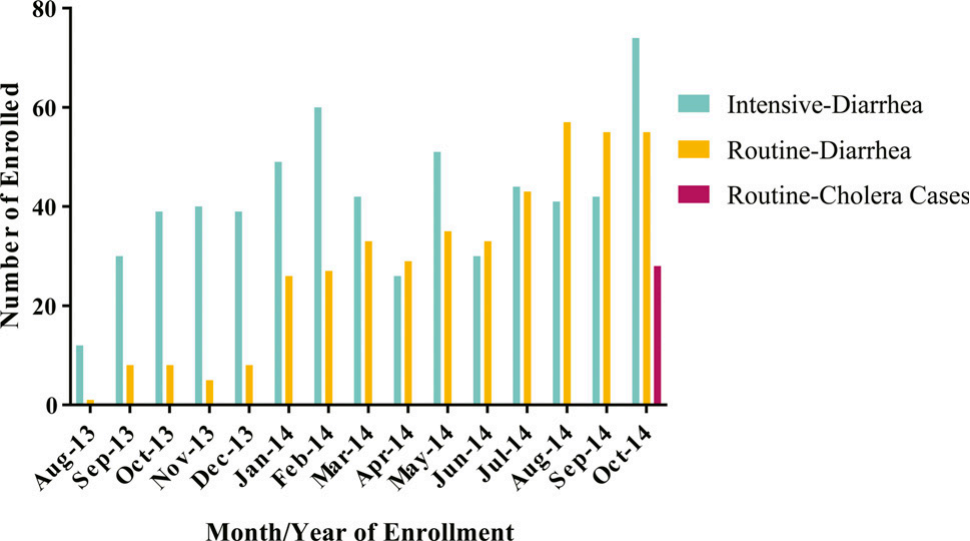
Monthly enrollments of diarrhea and cholera cases. The graph above shows the enrollment of diarrheal cases by month, stratified by surveillance type and cholera outcome.

**Figure 4.**
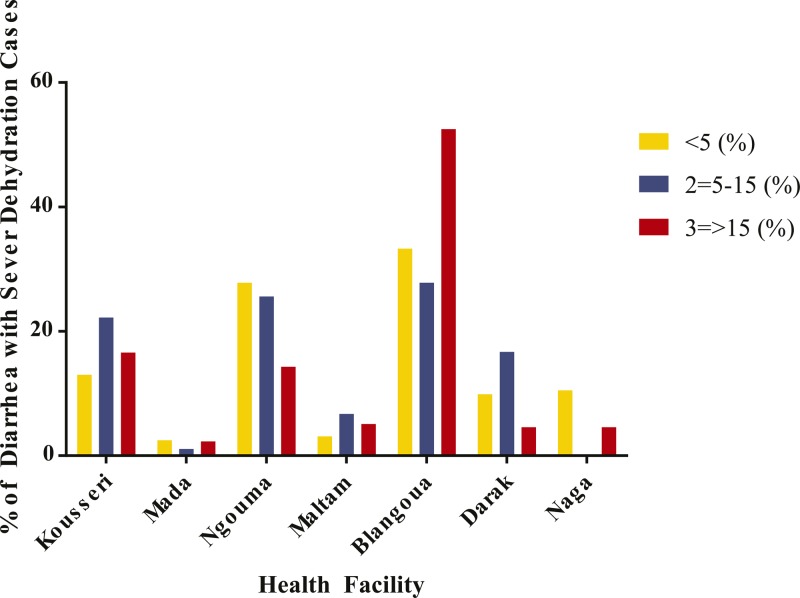
Percentage of diarrhea cases with severe dehydration stratified by age group across health facilities. The graph demonstrates the percentage of severe diarrheal cases enrolled at each health facility participating in the surveillance efforts. Blangoua had the highest numbers among all age groups.

From August 2013 through October 2014, a total of 1,042 patients were enrolled in the study among all seven study sites. Of these patients, 619 were enrolled in the study during intensive surveillance days, 423 were enrolled during routine surveillance days (Figure 3

). Figure 3 demonstrates that most cases of acute diarrhea presenting both during the intensive surveillance every 15 days and the routine surveillance were not caused by *V. cholerae*. Though cholera was not common, severe non-cholera diarrheal disease was prevalent in Blangoua health facilities (Figure 4

). Cholera was not detected in these health facilities until October 2014 when an outbreak was identified in Darak, Cameroon. The outbreak continued through October, with additional cases being detected in nearby Blangoua. In total, 32 PCR-confirmed cholera cases were enrolled: 30 patients had O1 *V. cholerae* and two had non-O1 cholera. Of these, 11 (34%) were children under 5 years of age and 21 (66%) were males (Table 2).

Of the 1,011 water samples obtained from the environmental sites for the seven health facilities during the study period, 244 were *V. cholerae* non-O1 and non-O139 positive, while none were *V. cholerae* O1 positive. (After this defined period of surveillance, in November 2014, one environmental sample was positive for *V. cholerae* O1 by dipstick.) The four types of water sources sampled included rivers, wells, sewage drains, and Lake Chad. Binomial regression analysis showed an increased risk of *Vibrio* in April, May, and July as compared with the risk in January (Table 3). At the facility level, there was a significantly increased likelihood of *Vibrio* detection at sites near Naga and Darak as compared with Kousseri. Finally, as compared with *Vibrio* detection in rivers, there is no significant increase of detection at sites along Lake Chad. However, a significant increase of *Vibrio* detection was seen in wells and sewage drains.

The sensitivity and specificity of the modified protocol for the Crystal VC dipstick was confirmed by PCR confirmation of 1,681 specimens; 673 clinical specimens were confirmed via PCR. Of the 641 clinical specimens that were PCR negative, 638 were dipstick negative, representing a Crystal VC dipstick specificity of 99.5% using the enriched methods (Table 4). All 1,011 environmental samples tested within the study time frame were both dipstick and PCR negative (100% specificity). There were an insufficient number of positive environmental samples to confirm sensitivity. The sensitivity, specificity, positive predictive value, and negative predictive value of PCR as compared with culture confirmation of the 507 clinical specimens confirmed using both methods was 90.9%, 99.3%, 95.2%, and 98.6%, respectively.

## Discussion

The use of simplified laboratory and epidemiological surveillance methodologies in this study enabled rapid identification of cases during an outbreak while significantly reducing any false-positive test results. This study is also the first to demonstrate the use of the enriched Crystal VC dipstick method in a remote setting. The results of the dipstick test were confirmed by a combination of culture and PCR for 673 clinical and 1,011 environmental samples, demonstrating a specificity of 99.5% and 100%, respectively. This is significantly higher than the specificity levels reported for direct use of the dipstick in stool samples (49–79%).^7^–^9^ These data validate the use of the enriched dipstick method to improve specificity as initially demonstrated in the hospital setting in Bangladesh^10^,^12^,^13^ and Mozambique.^11^ This is the first study to demonstrate the use of this enriched method in remote and rural settings with limited laboratory capacity.

This study presents the novel use of filter paper for environmental and stool sample preservation for molecular screening. This method proved to be a low-cost and low-maintenance diagnostic for the field setting, eliminating the need for culture confirmation and laboratory reagents. Although extensive training of laboratory personnel was previously required to conduct microbiological testing, this method enables non-laboratory personnel to implement the technique in a field setting. Furthermore, the filter paper does not need cold chain for preservation and is considered non-biohazardous after drying, thereby eliminating transportation issues.

This study is the first to investigate a simplified epidemiological and laboratory surveillance methodology for use in remote and rural settings and to successfully implement a sustained clinical and environmental surveillance of cholera in the FNC, particularly during a period of insecurity. Although the surveillance approach was modeled after a similar methodology used in Bangladesh,^4^ the methodology applied in Cameroon was further simplified by the use of the enriched dipstick test, the use of a low-cost gauze filtration device for environmental sampling, and the preservation of dried, enriched specimens on filter paper for PCR confirmation of initial results. In 2014, after more than 1 year of surveillance, the first cholera cases were identified during study surveillance activities in Darak and Blangoua health districts, and the cases were rapidly confirmed using the study-specific simplified laboratory methodologies. This enabled rapid response and interventions to limit the spread of the disease. Thus, not only did these findings contribute to the assessment of a surveillance strategy appropriate for remote and vulnerable settings, but it also demonstrated the capability of this method for early outbreak detection.

The surveillance results for the first year of this study did not confirm our initial hypothesis that there is an environmental reservoir for cholera in Lake Chad that leads to occasional cases. Nor did the regression analysis reveal a significant seasonal trend in *Vibrio* presence in the environmental sites. However, this report is based only on the first year of surveillance. Given that outbreaks in the study area were only in the beginning stages at the conclusion of this first report, continued surveillance over a longer period is needed.

There are several limitations to this study, the most important being that the study site is situated in an area that continues to struggle with safety and security issues as a result of terrorist activities. Safety issues resulted in the loss of data in some study areas, particularly in Darak, which is located on an island in Lake Chad. The presence of nontoxigenic *V. cholerae* O1 in the environmental sources during the early months of 2014 in Darak may have been an early warning sign for the area. Unfortunately, the team was unable to maintain regular surveillance, leading to an incomplete analysis of events before the toxigenic *V. cholerae* O1 outbreak in the fall of 2014. The conventional culture techniques were conducted on 507 samples in this study with only one culture positive/PCR negative sample. This false-negative PCR specimen, as well as many other samples, was collected by a single health worker who received only one abbreviated day of training amid the outbreak. The previously trained health-care workers at the site had fled the area before the outbreak because of mounting insecurity in the area. Although culture was completed at the central reference laboratory, the initial dipstick testing and filter paper preservation were completed at the site on case presentation. This highlights the simplicity of the techniques and ease of implementation in even the most challenging setting. There were no clinical cases identified in our study area before October 2014. This insufficient amount of positive results prevented us from establishing an accurate sensitivity calculation for the enriched dipstick methodology. Finally, only *V. cholerae* was confirmed clinically and, therefore, we were unable to further characterize the cases of non-cholera diarrhea enrolled in the study.

In conclusion, the first year of this surveillance study demonstrates the successful use and implementation of low-cost, simplified epidemiological and laboratory methodologies for surveillance in remote, rural, or vulnerable settings. The use of an enriched dipstick protocol was successfully applied in a field setting with improved specificity. Basic technologies, such as the use of gauze filtration rather than more expensive filtration methods, decrease supply and logistics costs and allow for important environmental surveillance to provide information about the burden of cholera in previously undescribed areas. Finally, the novel application of dried filter paper methodology to cholera DNA preservation demonstrates a simplified method for assessment for *Vibrio* presence in stool and environment.

## Figures and Tables

**Table 1:** Primers for PCR assays

Primer name	Sequence	Amplicon (bp)	Reference
UtoxF	GASTTTGTTTGGCGYGARCAAGGTT	–	18
VptoxR	GGTTCAACGATTGCGTCAGAAG	297	
VctoxR	GGTTAGCAACGATGCGTAAG	640	18
VvtoxR	AACGGAACTTAGACTCCGAC	435	
CtxA-F	CTCAGACGGGATTTGTTAGGCACG	302	19
CtxA-R	TCTATCTCTGTAGCCCCTATTACG		
OmpW-F	CACCAAGAAGGTGACTTTATTGTG	588	19
OmpW-R	GAACTTATAACCACCCGCG		
O1F2-1	GTTTCACTGAACAGATGGG	192	20
O1R2-2	GGTCATCTGTAAGTACAAC		
O139F2	AGCCTCTTTATTACGGGTGG	449	20
O139R2	GTCAAACCCGATCGTAAAGG		
6968 GC (V6/V8)	5′–AACGCGAACCTTAC–3′	457	21
L1401	3′–GCGTGTGTACAAGACCC–5′		

PCR = polymerase chain reaction.

**Table 2:** Clinical surveillance PCR results

Age group	No cholera (%)			Cholera (%)		
	Male	Female	Total	Male	Female	Total
< 5	116 (34)	95 (32)	211 (33)	8 (28)	3 (27)	11 (34)
5–15	67 (20)	54 (18)	121 (19)	10 (48)	2 (18)	12 (38)
> 15	158 (46)	151 (50)	309 (48)	3 (14)	6 (55)	9 (28)
Total	341 (53)	300 (47)	641	21 (66)	11 (34)	32

PCR = polymerase chain reaction.

**Table 3:** GEE results: *Vibrio* non-O1 detection

Factors	OR (95% CI)	*P* value
Month (January)	Ref	–
March	2.5 (1.9, 15.6)	0.001
April	4.8 (1.5, 14.7)	0.007
May	2.6 (0.9, 7.6)	0.091
June	1.9 (0.6, 6.2)	0.307
July	3.9 (1.2, 12.1)	0.021
Water source (river)	Ref	–
Well	3.0 (1.2, 7.3)	0.016
Sewage drain	10.4 (2.8, 38.1)	< 0.001
Lake Chad	0.4 (0.1, 2.5)	0.298
Facility (Kousseri)	Ref	–
Blangoua	1.2 (0.3, 5.4)	0.837
Darak	9.7 (2.4, 38.3)	0.001
Naga	14.7 (3.8, 57.6)	< 0.001

CI = confidence interval; GEE = generalized estimating equation; OR = odds ratio.

**Table 4:** PPV and NPV, sensitivity and specificity of enriched Crystal VC dipstick as compared with the gold standard of PCR

Enriched dipstick method	Clinical samples		Total
	PCR positive	PCR negative	
Dipstick positive	25	3	28
Dipstick negative	7	638	645
Total	32	641	673
		Estimate (%)	95% CI
Sensitivity		89.3	71.8–97.7
Specificity		98.9	97.8–99.6
PPV		78.1	60.0–90.7
NPV		99.5	98.6–99.9

CI = confidence interval; NPV = negative predictive value; PCR = polymerase chain reaction; PPV = positive predictive value.
